# The role of l-serine and l-threonine in the energy metabolism and nutritional stress response of *Trypanosoma cruzi*

**DOI:** 10.1128/msphere.00983-24

**Published:** 2025-03-05

**Authors:** Mayke Bezerra Alencar, Richard Marcel Bruno Moreira Girard, Marcell Crispim, Carlos Gustavo Baptista, Marc Biran, Frederic Bringaud, Ariel Mariano Silber

**Affiliations:** 1 Laboratory of Biochemistry of Trypanosomatids-LaBTryps, Department of Parasitology, Institute of Biomedical Science II-ICB II, University of São Paulo-USP, São Paulo, São Paulo, Brazil; 2 Microbiologie Fondamentale et Pathogénicité (MFP), UMR 5234, Univ. Bordeaux, CNRS, Bordeaux, France; 3 Centre de Résonance Magnétique des Systèmes Biologiques (CRMSB), UMR 5536, Univ. Bordeaux, CNRS, Bordeaux, France; University at Buffalo—Downtown Campus, Buffalo, New York, USA

**Keywords:** *Trypanosoma cruzi*, amino acid metabolism, transport, bioenergetics, nutritional stress

## Abstract

l-Serine and l-threonine have versatile roles in metabolism. In addition to their use in protein synthesis, these amino acids participate in the biosynthesis pathways of other amino acids and even phospholipids. Furthermore, l-serine and l-threonine can be substrates for a serine/threonine dehydratase (Ser/ThrDH), resulting in pyruvate and 2-oxobutyrate, respectively, thus being amino acids with anaplerotic potential. *Trypanosoma cruzi*, the etiological agent of Chagas disease, uses amino acids in several biological processes: metacyclogenesis, infection, resistance to nutritional and oxidative stress, osmotic control, etc. This study investigated the import and metabolism of l-serine, l-threonine, and glycine in *T. cruzi*. Our results demonstrate that these amino acids are transported from the extracellular environment into *T. cruzi* cells through a saturable transport system that fits the Michaelis-Menten model. Our results show that l-serine and l-threonine can sustain epimastigote cell viability under nutritional stress conditions and stimulate oxygen consumption, maintaining intracellular ATP levels. Additionally, our findings indicate that serine plays a role in establishing the mitochondrial membrane potential in *T. cruzi*. Serine is also involved in energy metabolism via the serine-pyruvate pathway, which stimulates the production and subsequent excretion of acetate and alanine. Our results demonstrate the importance of l-serine and l-threonine in the energy metabolism of *T. cruzi* and provide new insights into the metabolic adaptations of this parasite during its life cycle.

**IMPORTANCE:**

*Trypanosoma cruzi*, the parasite responsible for Chagas disease, impacts 5–6 million individuals in the Americas and is rapidly spreading globally due to significant human migration. This parasitic organism undergoes a complex life cycle involving triatomine insects and mammalian hosts, thriving in diverse environments, such as various regions within the insect’s digestive tract and mammalian cell cytoplasm. Crucially, its transmission hinges on its adaptive capabilities to varying environments. One of the most challenging environments is the insect’s digestive tract, marked by nutrient scarcity between blood meals, redox imbalance, and osmotic stresses induced by the triatomine’s metabolism. To endure these conditions, *T. cruzi* has developed a remarkably versatile metabolic network enabling it to metabolize sugars, lipids, and amino acids efficiently. However, the full extent of metabolites this parasite can thrive on remains incompletely understood. This study reveals that, beyond conventional carbon and energy sources (glucose, palmitic acids, proline, histidine, glutamine, and alanine), three additional metabolites (serine, threonine, and glycine) play vital roles in the parasite’s survival during starvation. Remarkably, serine and threonine directly contribute to ATP production through a serine/threonine dehydratase enzyme not previously described in *T. cruzi*. The significance of this metabolic pathway for the parasite’s survival sheds light on how metabolic networks aid in its endurance under extreme conditions and its ability to thrive in diverse metabolic settings.

## INTRODUCTION

*Trypanosoma cruzi,* the etiological agent of Chagas disease, transitions between different environments of different hosts, such as the mammalian host-cell cytoplasm, the mammalian blood, and different regions of the reduviid insect’s digestive tube (midgut and rectum lumen) during its life cycle. To survive in these environments, *T. cruzi* reprograms its metabolism, being able to metabolize various carbon/energy sources according to their abundance (1–5). In this regard, this parasite can switch between the consumption of carbohydrates, amino acids, and fatty acids (6–10).

Among the available nutrients in most environments colonized by *T. cruzi,* l-serine (l-Ser), l- threonine (l-Thr), and glycine (Gly) are almost omnipresent (11–16). Despite this, in-depth studies of the metabolism of these amino acids in this parasite are scarce. It has been determined that l-Ser triggers the production of CO_2_ , indicating its possible use in bioenergetics (17). In the related organism *Trypanosoma brucei,* the participation of l-Thr in the mitochondrial metabolism was well demonstrated (18–20). Although there is no evidence that Gly participates in energy metabolism for any of these organisms, the carbons of Gly might contribute to the formation of l-Ser through the reversible activity of serine hydroxymethyltransferase (SHMT) (21, 22). Therefore, it is worth investigating its possible participation in the parasite’s bioenergetics. To summarize, despite the demonstrated potential of these amino acids to trigger ATP biosynthesis, their consumption and metabolism in *T. cruzi* remain almost unexplored. This work explores the uptake of l-Ser, l-Thr, and Gly and their role in the parasite’s bioenergetics and nutritional stress (NS) resilience.

## RESULTS

### *T. cruzi* epimastigotes transport l-Ser, l-Thr, and Gly from the extracellular medium

**Fig 1:**
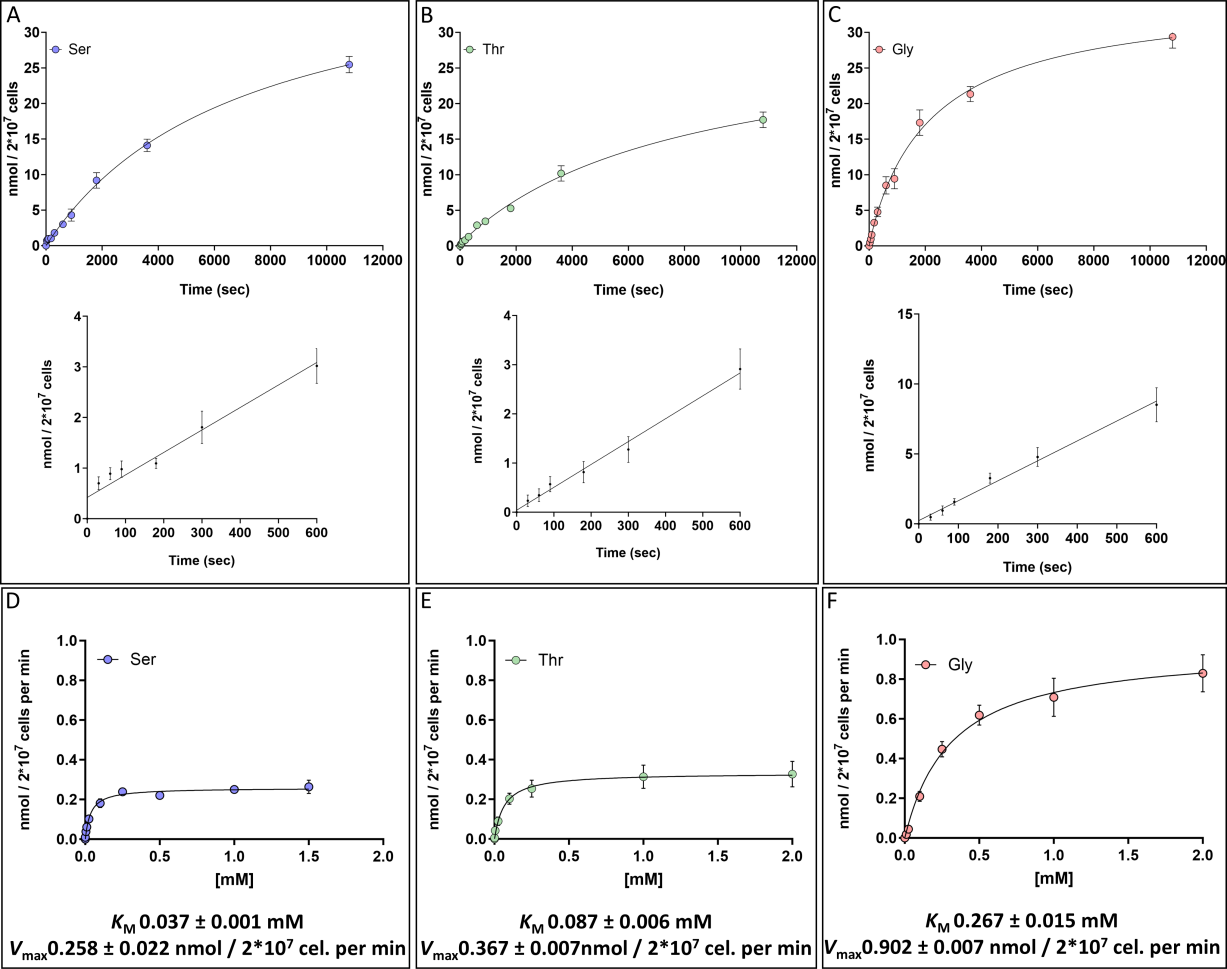
Uptake of l-Ser, l-Thr, and Gly in *T. cruzi* epimastigote as a function of time and concentration. (**A–C**) Time-course transport of 5 mM l-Ser, l-Thr, and Gly, respectively. (**D–F**) Uptake of l-Ser, l-Thr, and Gly, respectively, as a function of their concentration. The insets represent the adjustment of incorporation as a function of time to a linear function. The text below the graphs gives the values of the kinetic parameters calculated by the Michaelis-Menten model. *r*^2^ values of 0.96, 0.95, and 0.94 for l-Ser, l-Thr, and Gly, respectively. All data were shown as mean  ±  SD (*n* = 3). All experiments were replicated three times or more in three biological replicates.

The initial step for l-Ser, l-Thr, and Gly metabolism is its uptake, which was initially measured as a function of time. For this, the parasites were incubated with each radioactively traced amino acid at a 5 mM concentration, assuming that this concentration was saturating for each transport system. The obtained data for each analyzed metabolite fit to an exponential decay function (l-Ser: *r*^2^ = 0.99, l-Thr: *r*^2^ = 0.98, and Gly: *r*^2^ = 0.98), as expected for saturable protein-mediated transport systems (Fig. 1A through C). The uptake of the three amino acids was shown to be near-linear until 10 min (*r*^2^ = 0.92, *r*^2^ = 0.94, and *r*^2^ = 0.95 for l-Ser, l-Thr, and Gly, respectively [insets in Fig. 1A through C]), which allowed us to set the time window to measure the initial velocity (*V*_0_) of l-Ser, l-Thr, and Gly uptake in 3 min.

To analyze the kinetics for the transport of each amino acid, epimastigotes were incubated with different concentrations of l-Ser, l-Thr, or Gly, *V*_0_ was measured, and the data were robustly fitted to the Michaelis-Menten kinetic function (*r*^2^ = 0.96, 0.95, and 0.94 for l-Ser, l-Thr, and Gly, respectively). The obtained values for *K*_*M*_ and *V*_max_ for the three substrates (Fig. 1D through F) were 37 ± 0.001 µM, 87 ± 0.006 µM, and 267 ± 0.015 µM, and 0.258 ± 0.022 nmol min^−1^ 2 × 10^7^ cells, 0.367 ± 0.007 nmol min^−1^ 2 × 10^7^ cells, and 0.902 ± 0.007 nmol min^−1^ 2 × 10^7^ cells for l-Ser, l-Thr, and Gly, respectively (Fig. 1D through F).

Then, the possibility of l-Ser, l-Thr, and Gly sharing their transport system was investigated by cross-competition assay. The uptake of each amino acid was measured by using each labeled metabolite at its *K*_*M*_ concentration (37, 87, and 260 µM for l-Ser, l-Thr, and Gly, respectively) in the presence of the potentially competing amino acid at a concentration corresponding to 10-fold the *K*_*M*_ value (370, 870, and 2,600 µM for l-Ser, l-Thr, and Gly, respectively) (Table 1). The uptake of l-Ser was diminished by 50% and 45%, respectively, by l-Thr and Gly, while the uptake of l-Thr was reduced by 82% and 42%, respectively, by l-Ser and Gly, and the transport of Gly was inhibited by 83% and 58%, respectively, by l-Ser and l-Thr. Our findings show that l-Ser, l-Thr, and Gly compete with each other, supporting the hypothesis of a shared transport system for these three amino acids. As expected, inhibition varies according to the competitor’s affinity for the transport system. l-Ser uptake was set as a proxy to characterize the activity for all three. Using the same conditions, the ability of other amino acids to impair l-Ser transport was assessed. While structurally related amino acids, such as d-Ser, l-Cys, d-Ala, and l-Ala, resulted in partial inhibition of l-Ser transport, structurally dissimilar amino acids, such as branched-chain amino acids (BCAA; l-leucine, l-isoleucine, and l-valine) (23), l-histidine (His) (24), and l-phenylalanine did not significantly affect it, as expected (Table 1).

**TABLE 1:** Percentage of inhibition in transport observed in cross-competition assay^*a*^

Competitor	% of inhibition of l-Ser uptake	% of inhibition of l-Thr uptake	% of inhibition of Gly uptake
l-Ser	–^*b*^	82.7 ± 1.0	83.2 ± 1.4
d-Ser	39.65 ± 1.02	22.5 ± 2.3	31 ± 3.6
Gly	45.01 ± 5.50	42.3 ± 3.1	–
l-Thr	50 ± 3.48	–	58 ± 2.8
l-Ala	21.90 ± 4.02	–	–
d-Ala	25.12 ± 1.38	–	–
l-Cys	30.88 ± 7.24	–	–
l-His	No inhibition	–	–
l-Leu	No inhibition	–	–
l-Phe	No inhibition	–	–

^*a*^ Cross-competition assay between l-Ser, l-Thr, and Gly**:** transport inhibition of each amino acid was determined by adding an excess (10 × *K*_*M*_ value) for each amino acid tested. All data were shown as mean ± SD (*n* = 3). All experiments were replicated three times or more in three biological replicates.

^*b*^ –, not done.

### Determination of the driving force for l-Ser uptake

**Fig 2:**
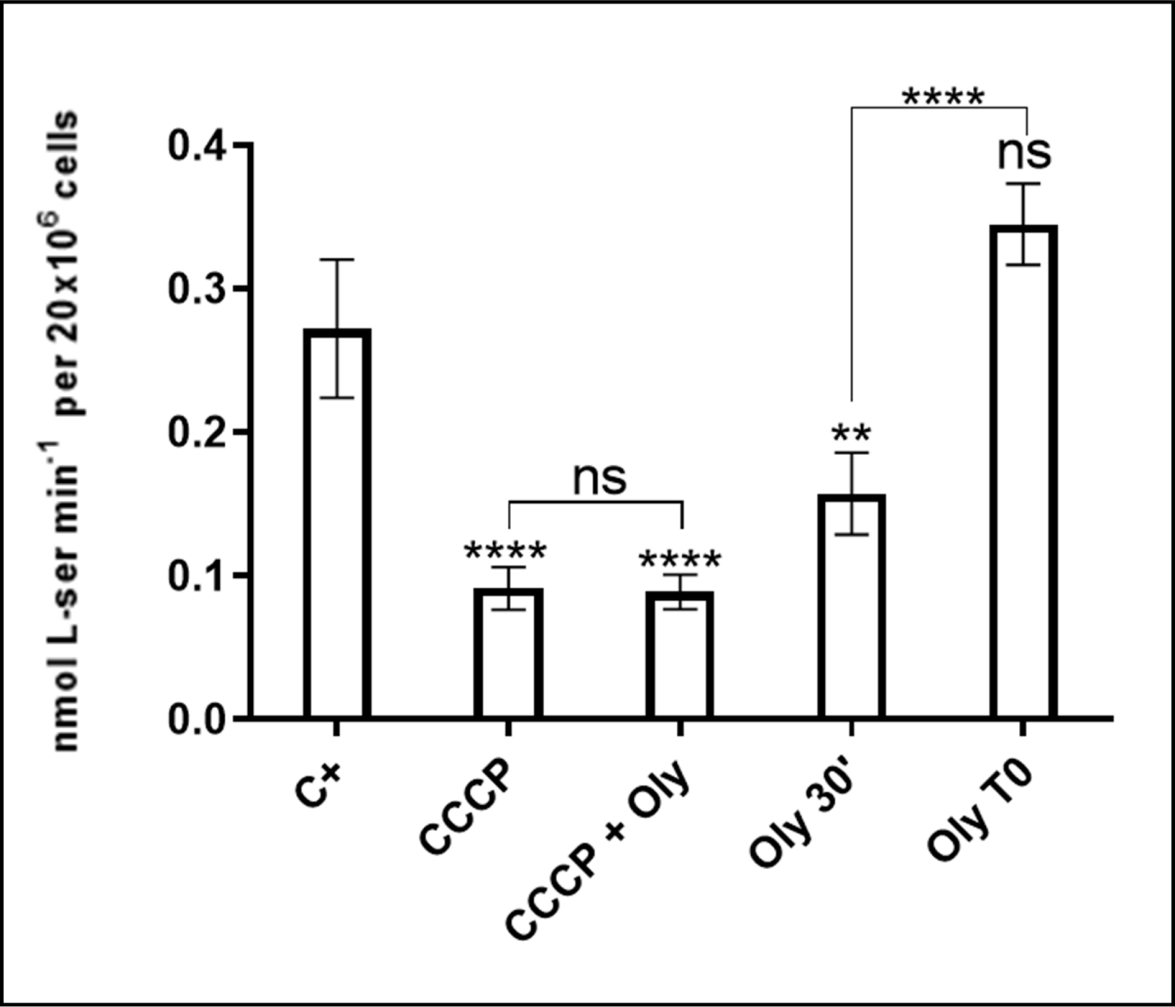
Effect of oligomycin A (Oly) and CCCP on l-Ser uptake: the dependence of l-Ser transport on intracellular ATP levels (Oly 30′) and the H^+^ gradient (CCCP) were assessed. CCCP can rapidly trigger ATP hydrolysis by mitochondrial ATPase to reestablish the H^+^ gradient, leading to ATP depletion in the cell. To distinguish between these phenomena, the parasites were incubated with CCCP in the presence and absence of Oly (CCCP + Oly). To control for non-specific (off-target) inhibition of the transport system by oligomycin A, we added it without pre-incubation (Oly T0). All data were shown as mean  ±  SD (*n* = 3). All experiments were replicated three times or more in three biological replicates. Statistical analysis was performed using one-way ANOVA with Tukey’s post-test (****P < 0.0001; **P: 0.002).

To investigate the effect of intracellular ATP levels on this transport system, l-Ser uptake was measured in parasites treated for 30 min with oligomycin A. This known F_O_-ATP synthase inhibitor produces intracellular ATP depletion in *T. cruzi* (Fig. S1A) (23) or without treatment (control). A control group treated with oligomycin A immediately before measuring the l-Ser uptake was included to discard an off-target effect of oligomycin A on the l-Ser transport. Cells pre-incubated with oligomycin A for 30 min showed a decrease in l-Ser uptake of approximately 40.3% ± 13%. In contrast, l-Ser uptake levels were unaffected without pre-incubation compared with the untreated control. To determine whether the transport system depended on the proton gradient across the plasma membrane, the *V*_0_ of l-Ser incorporation in the presence of the protonophore carbonyl cyanide *m*-chlorophenyl hydrazone (CCCP) was measured. CCCP has two known effects on cells: (i) it collapses the proton gradient of the whole cell (Fig. S1B), and (ii) it depletes intracellular ATP levels by reversing the mitochondrial F_1_F_o_-ATP synthase reaction (25–28). To distinguish these two effects on the transport assay, the l-Ser transport was also measured in the presence of CCCP supplemented with oligomycin A (to prevent ATP hydrolysis by reversal of the F_1_F_o_-ATP synthase activity). The disruption of the proton gradient significantly diminished the transport activity compared to the positive control (C+), indicating a proton gradient-dependent process (Fig. 2 and Table 2).

**TABLE 2:** Inhibition of l-Ser uptake in the presence of CCCP and oligomycin A^*c*^

Treatment	% of l-Ser uptake inhibition
10 µM CCCP	63.8 ± 9
10 µM CCCP + 0.5 µg/µL Oly^*b*^	66.3 ± 0.2
0.5 µg/µL Oly 30′	40.3 ± 13
0.5 µg/µL Oly T0	nd^*a*^

^*a*^ nd, not detected.

^*b*^ Oligomycin A.

^*c*^ All data were shown as mean ± SD (*n* = 3). All experiments were replicated three times or more in three biological replicates.

### Thermodynamic analysis of l-Ser uptake

**Fig 3:**
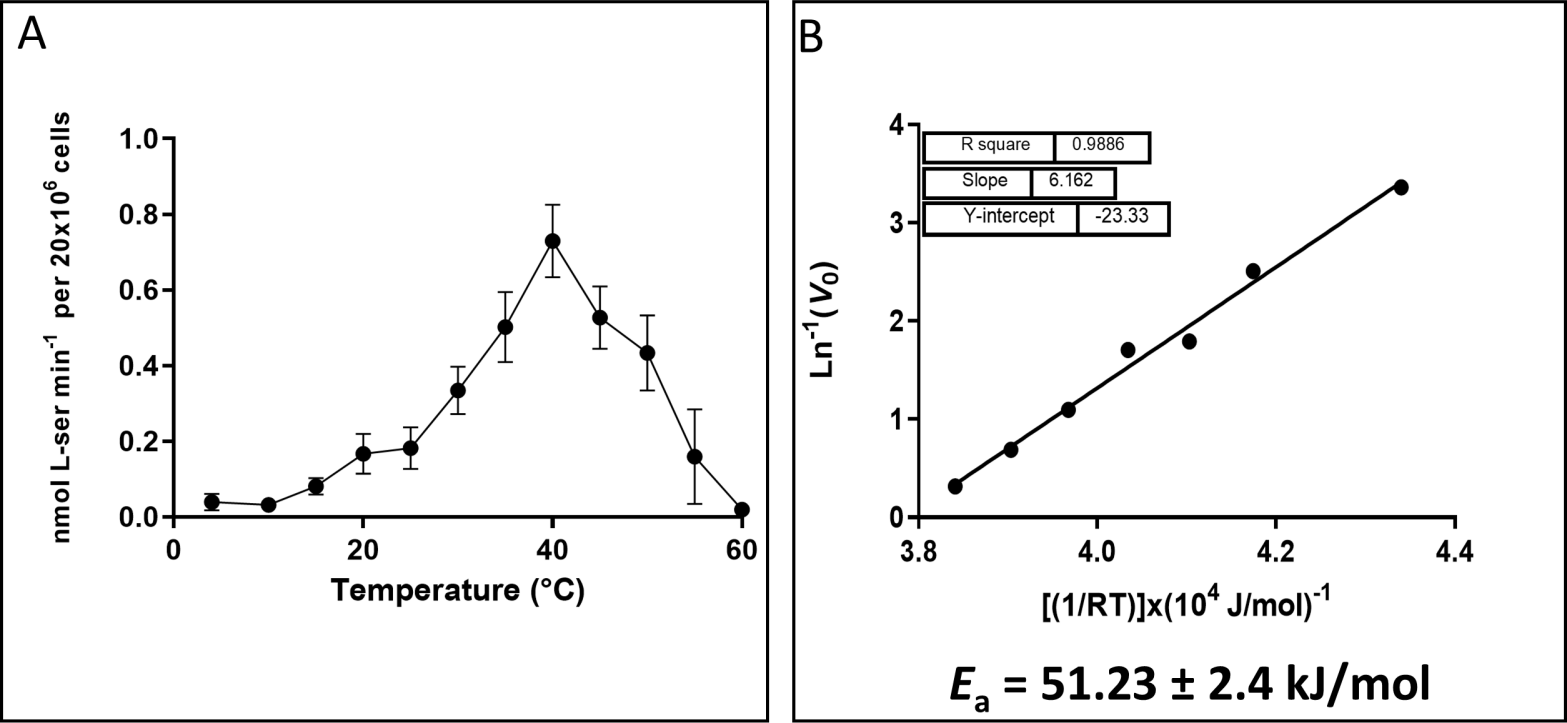
Effect of temperature on l-Ser uptake. (**A**) The *V*_0_ was measured at saturating concentrations of l-Ser after 3 min of uptake at specific temperatures. (**B**) An Arrhenius plot was created by performing a linear fit between the *V*_0_ values measured at temperatures ranging from 4°C to 40°C. All data were shown as mean ± SD (*n* = 3). All experiments were replicated three times or more in three biological replicates.

To assess the effect of temperature on l-Ser uptake, the *V*_max_ was measured at different temperatures ranging between 4°C and 60°C, which showed a maximum at 40°C. In addition, under these conditions, the changes in *V*_max_ in the exponential region of the curve were utilized to calculate a *Q*_10_ of 1.948. An Arrhenius linear representation (*R*^2^: 0.988) was used to calculate an activation energy (*E*_*a*_) of 51.2 ± 2.4 kJ/mol (Fig. 3A and B). Additionally, from the Arrhenius equation, it was possible to calculate the transporter kinetic turnover, which was used to estimate the number of transporters as 0.099 attomol/cell (see Text S1), equivalent to approximately 6 × 10^4^ active sites per cell.

### Regulation of l-Ser uptake in the different developmental stages of the parasite

**Fig 4:**
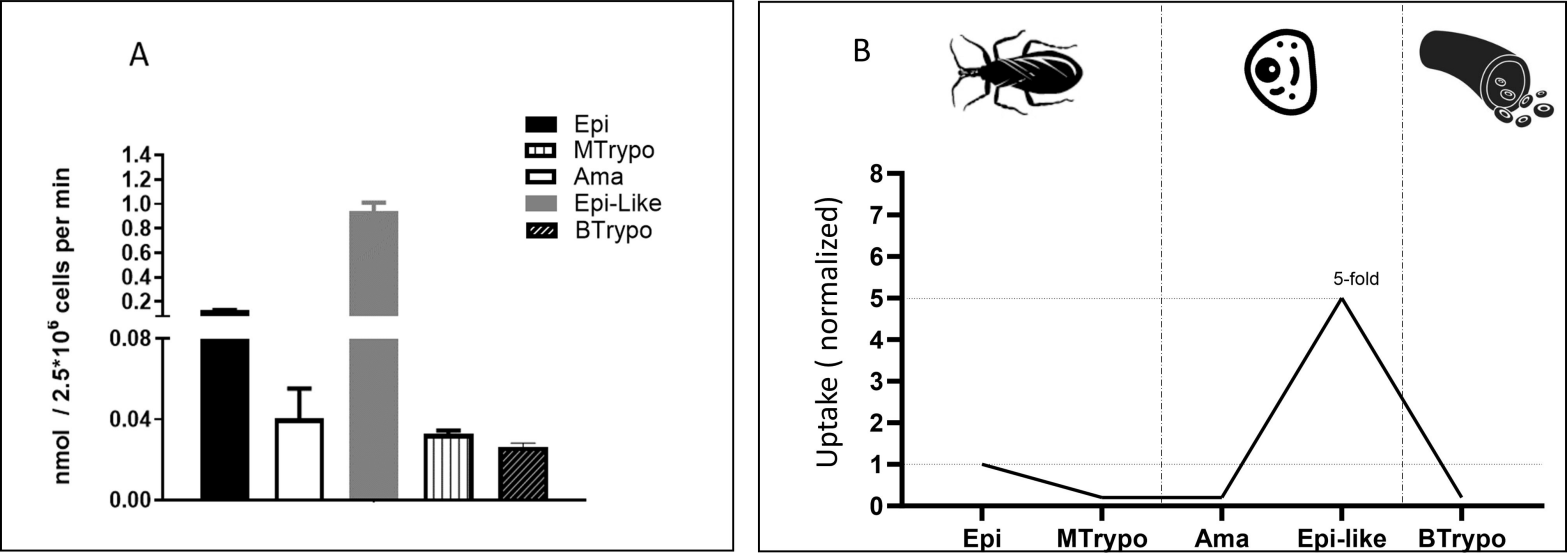
Transport of Ser in different life-cycle stages of *T. cruzi*. (**A**) Transport of l-Ser. (**B**) Transport of l-Ser (normalized). All data were shown as mean  ±  SD (*n* = 3). All experiments were replicated three times or more in three biological replicates. The panel on the right represents the normalized transport rates, considering the transport measured in epimastigotes as 100%.

To assess the possible regulation of the l-Ser uptake in the different developmental stages of the parasite, the activity was measured in the intracellular stages (amastigote—Ama and intracellular epimastigote—Epi-like) (29), the stages present in the insect vector (epimastigote—Epi and metacyclic trypomastigote—MTrypo), and those present in the bloodstream of vertebrates (cell-derived trypomastigote—BTrypo). The obtained results show that l-Ser transport varies among the parasite’s life-cycle stages. Interestingly, the intracellular Epi-like stage captured approximately five times more l-Ser from the medium than Epi forms, while MTrypo and BTrypo captured five times less than epimastigote (Fig. 4A and B).

### Participation of l-Ser, l-Thr, and Gly in the resistance to nutritional stress in *T. cruzi* epimastigotes

**Fig 5:**
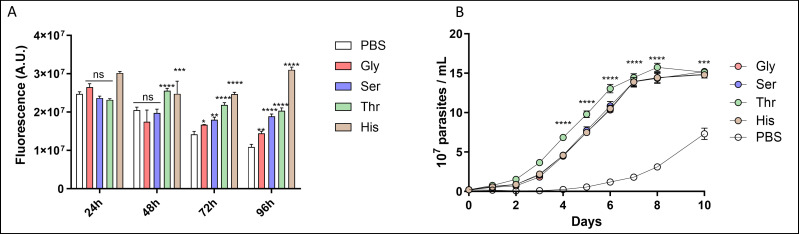
(**A**) Viability assay of *T. cruzi* epimastigotes as a function of nutritional stress: viability was measured by the irreversible reduction of resazurin to resorufin. l-His (5 mM) was used as a positive control, and no exogenous carbon source (PBS) was used as a negative control. (**B**) Recovery assay of *T. cruzi* epimastigotes (as described in Materials and Methods) after NS conditions: the proliferation profile was evaluated after 72 h of NS in the presence or absence (only PBS) of exogenous carbon sources, 5 mM His (positive control) and 5 mM Gly, l-Ser, and l-Thr. Calibration curves were performed using known parasite densities. As a negative control, PBS without exogenous carbon sources was used. All data were shown as mean ± SD (*n* = 3). All data were compared with the PBS control and were replicated three times or more in three biological replicates. Two-way ANOVA with Tukey’s post-test was used for statistical analysis: **P < 0.0029; ***P < 0.0007; and ****P < 0.0001.

To initially investigate the role of the analyzed amino acids on the resistance to NS, the cells were incubated in PBS supplemented or not (as a negative control) with 5 mM of the amino acids under study or l-His as a control for survival (24). The use of l-His as a positive control was chosen based on well-established bioenergetic, viability, mitochondrial inner membrane potential, and recovery from starvation data. l-His performed similarly to the LIT medium in terms of bioenergetic parameters and viability, with the advantage of a more controlled exogenous medium (24, 30). After 48 h, only l-Thr succeeded in maintaining cell viability at the level of positive control. However, during longer NS incubations (72 and 96 h), Gly and l-Ser could sustain viability significantly better than the PBS control (Fig. 5A). As survival does not necessarily imply the cells’ capacity to resume proliferation, this possibility was investigated by performing a proliferation recovery experiment following NS. Our results showed that l-Ser, l-Thr, and Gly can maintain the proliferative capacity of epimastigote forms when re-incubated in rich LIT medium after arrest induced by NS, at similar levels as the positive control (l-His) (Fig. 5B). Taken together, these results indicate that all three metabolites can protect the cells from NS, with l-Thr being more efficient than l-Ser and Gly.

### Role of serine and threonine on epimastigote bioenergetics

**Fig 6:**
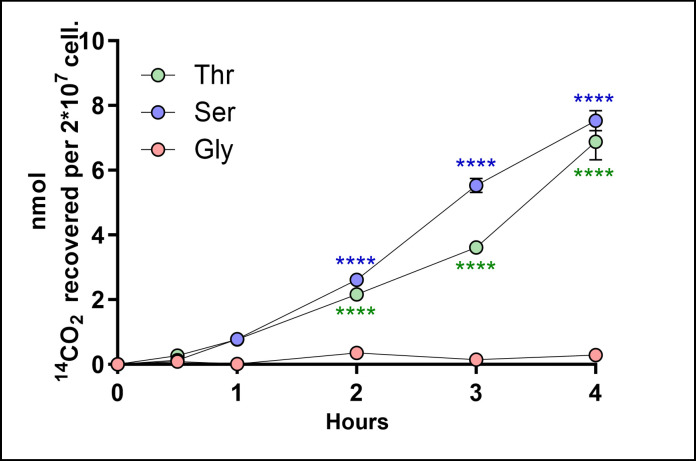
Production of ^14^CO_2_ by breaking down l-Ser, l-Thr, and Gly in *T. cruzi* epimastigote. To trap the produced ^14^CO_2_, pieces of Whatman filter soaked in 2 M KOH were placed on the top of the tubes where the parasites were incubated. The filters were recovered and mixed with a scintillation cocktail, and the K_2_^14^CO_3_ trapped on the paper was measured by using a scintillation counter (as described in Materials and Methods). The data were shown as mean ± SD (*n* = 3). All data were compared with the values obtained for Gly, and the experiments were replicated three times or more in three biological replicates. Two-way ANOVA with Tukey’s post-test was used for statistical analysis: ****P < 0.0001.

In most organisms, l-Ser and l-Thr feed the tricarboxylic acid cycle (TCA cycle), and they are broken down into CO_2_ (18, 20, 31–39). To investigate this possibility, epimastigote forms were incubated with l-[3-^14^C]Ser, l-[U-^14^C]Thr, and [U-^14^C]Gly to assess the ^14^CO_2_ production from these metabolites. The data show that l-Ser and l-Thr, but not Gly, were catabolized to CO_2_ (Fig. 6).

To track how much of the l-Ser, l-Thr, or Gly taken up by the cells was oxidized to CO_2_, the relationship between each metabolite uptake and CO_2_ production fluxes was calculated. After 3 h of incubation, 65.1% of the radiolabeled carbons of l-Ser and 21% of the radiolabeled carbons of l-Thr transported into the cells were recovered as ^14^CO_2_. At the same time, no CO_2_ from [U-^14^C]Gly was detected (Table 3). According to these data, l-Ser and l-Thr participate in mitochondrial-driven catabolism on the TCA cycle.

**TABLE 3:** Time-dependent ^14^CO_2_ generation by epimastigote form from l-[3^14^C]Ser, l-[U^14^C]Thr, and [U^14^C]Gly^*a*^

	Time (min)	^14^CO_2_ (nmol/1 × 10^7^ cell)	Uptake (nmol/1 × 10^7^ cell)	% recovered ^14^CO_2_^*d*^
l-Ser	30	0.24 ± 0.02^*b*^	9.18 ± 1.06	2.61
	60	2.67 ± 0.23^*b*^	14.11 ± 0.84	18.93
	180	16.59 ± 0.62^*b*^	25.47 ± 1.1	65.1
l-Thr	30	0.27 ± 0.1	5.28 ± 0.35	5.08
	60	0.81 ± 0.22	10.19 ± 1.04	7.99
	180	3.74 ± 0.22	17.72 ± 1.05	21.08
Gly	30	0.09 ± 0.07^*c*^	17.31 ± 1.73	0.52
	60	0.01 ± 0.02^*c*^	21.33 ± 1.02	0.07
	180	0.19 ± 0.38^*c*^	29.38 ± 1.02	0.65

^*a*^ All data were shown as mean ± SD (*n* = 3). All experiments were replicated three times or more in three biological replicates.

^*b*^ Considering that the only way for l-[3^14^C]Ser to be released as CO_2_ is through complete degradation in the TCA cycle, the obtained value was normalized by multiplying it by 3. In contrast, the values obtained for l-[U-^14^C]Thr or [U-^14^C]Gly have not been normalized since all carbon are ^14^C-labeled.

^*c*^ Considering the standard error, the percentage of ^14^CO_2_ produced from Gly was assumed to be residual and may have originated from the residual activity of the glycine cleavage complex.

^*d*^ To assess the comparative release of carbon as CO_2_, the measured values were normalized against the quantity of the transported amino acid over the identical incubation duration detected in Fig. 1A through C.

**Fig 7:**
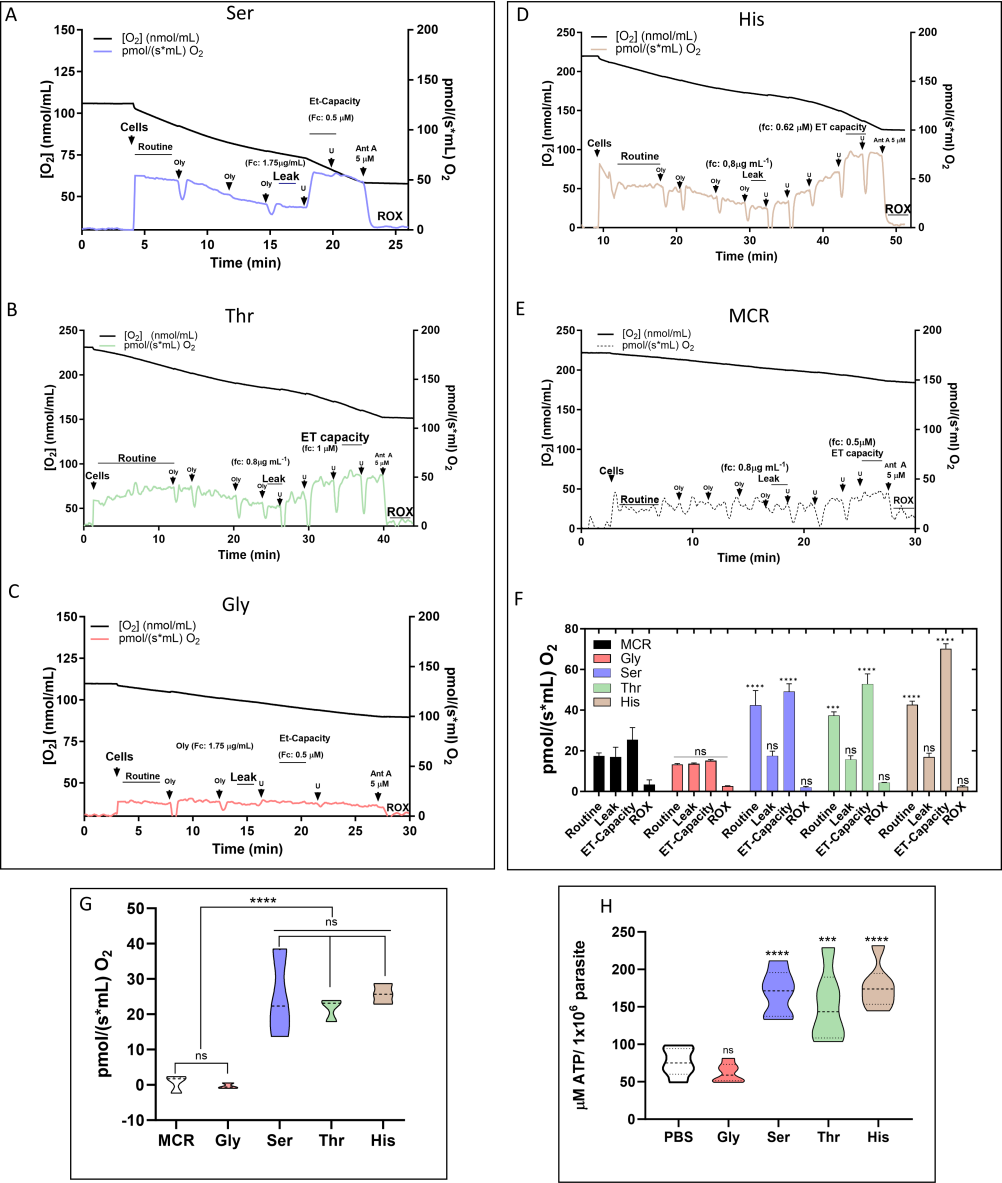
High-resolution respirometry in *T. cruzi* epimastigotes. Respiration rates were measured after 16 h of NS when the parasites were recovered for 3 h in the presence of a substrate. (**A–C**) Respiration rates after 3 h of incubation with 5 mM l-Ser, l-Thr, and Gly, respectively; (**D and E**) respiration rates after 3 h of incubation with 5 mM l-His and without exogenous carbon sources, respectively (MCR, mitochondrial cell respiration buffer). (**F**) Bar plot analysis of all data collected from respiration assay. (**G**) Free routine activity in epimastigotes recovered from NS with Gly, l-Ser, l-Thr, and l-His. The free routine activity was obtained by subtracting the respiratory rates measured after adding oligomycin A. The graph is representative of this subtraction, using the average of the slopes obtained with the amino acids and after inhibition of F_o_-ATP synthase with oligomycin A. (**H**) The parasites were recovered for 3 h in the presence (or not, negative control—only PBS) of 5 mM l-His (positive control) Gly, l-Ser, or l-Thr. ATP levels were measured by detecting luminescence in a coupled luciferase reaction. All data were shown as mean ± SD (*n* = 3). All experiments were replicated three times or more in three biological replicates. Two-way ANOVA with Tukey post-test was used for statistical analysis. ****P < 0.0001 and ***P < 0.001.

Previous observations suggest that at least l-Ser and l-Thr are involved in oxidative phosphorylation (OxPhos), so their participation in mitochondrial respiration was assessed. The cells were subjected to high-resolution oxygraphy, from which the respiration parameters were derived: routine respiration (R), rate of resting respiration of cells incubated with any metabolite; leak respiration (L), the remaining O_2_ consumption measured when the ATP synthase is inhibited; electron-transport capacity, obtained after uncoupling the mitochondria by the addition of an uncoupler; and residual respiration rate, obtained by the inhibition of complex III (40). l-Ser and l-Thr (but not Gly) were able to trigger O_2_ consumption (Fig. 7A through C) when compared with the negative control (Fig. 7E) and similarly to our positive control (Fig. 7D). Furthermore, when L was subtracted from R to obtain the free routine respiration (respiration rate directly related to the ATP biosynthesis by F_o_F_1_-ATP synthase) (41), l-Ser and l-Thr performed similarly to l-His (Fig. 7G). In the same conditions, the recovery of intracellular ATP levels after 16 h NS was measured by a luciferase assay. Parasites incubated with l-Ser and l-Thr exhibited a significant recovery of the intracellular ATP levels, comparable to those of parasites recovered with l-His (positive control) compared to the negative control (PBS). Interestingly, Gly could not restore intracellular ATP levels in accordance with the absence of CO_2_ production and oxygen consumption (Fig. 7H). Together, these results show the anaplerotic capacity of l-Ser and l-Thr, and thus, their ability to support ATP synthesis via OxPhos.

**Fig 8:**
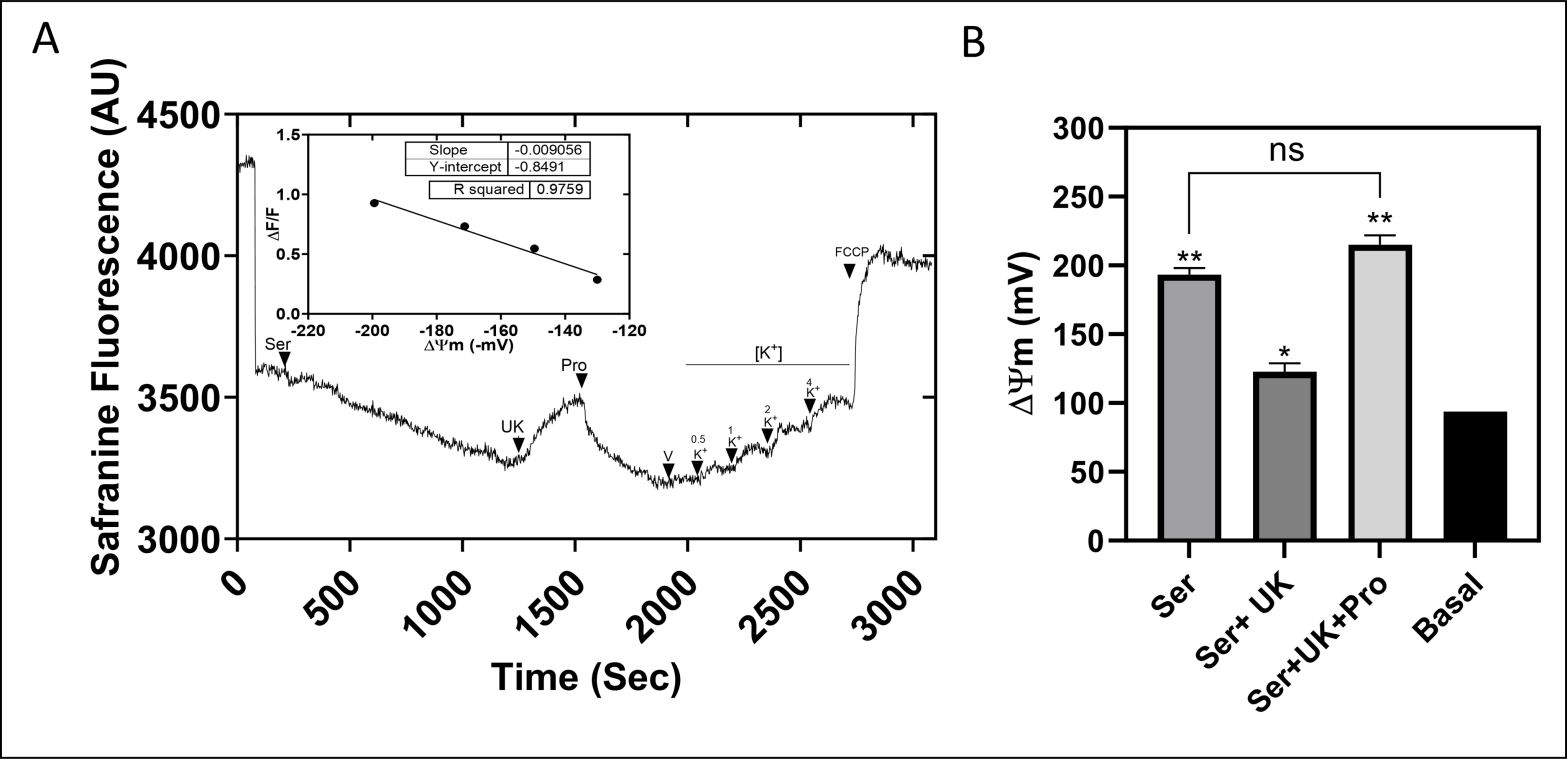
Quantification of ΔΨ_*m*_ in the presence of l-Ser and l-Pro. (**A**) Representative trace of the ΔΨ_*m*_ quantification experiment by fluorometry, using safranine O in the presence of l-Ser, UK-5099 (UK), and Pro (positive control). Inset: calibration curve performed with K^+^ in the presence of 5 nM valinomycin (V). (**B**) Quantification of ΔΨ_*m*_ under the previously mentioned conditions using the Nernst equation (see Materials and Methods). All data were shown as mean ± SD (*n* = 3). All experiments were replicated three times or more in three biological replicates. An unpaired *t* test was used. **P = 0.0011. *P = 0.0225.

Knowing that l-Ser stimulates both oxygen consumption and ATP biosynthesis, it is conceivable that this substrate could also contribute to establishing and maintaining the mitochondrial ΔΨ_*m*_ in *T. cruzi*. Given that l-Ser can form pyruvate through Ser/ThrDH, the possible relationship between ΔΨ_*m*_ generated by l-Ser and the mitochondrial pyruvate carrier (MPC) (42) was tested by adding 5 µM UK-5099, a specific inhibitor of the MPC (Fig. 8A). As expected, l-Ser was able to build up and maintain ΔΨ_*m*_ in epimastigotes. Consistent with the formation of pyruvate through Ser/ThrDH, the generated ΔΨ_*m*_ was reversed in the presence of UK-5099. To rule out a possible off-target effect of UK-5099 altering the capacity of building up ΔΨ_*m*_, 2 mM l-Pro, a metabolite that can directly feed electrons into the respiratory chain, was added as a control after UK 5099 (43, 44). As expected, the addition of l-Pro resulted in reestablishing the ΔΨ_*m*_. The quantification of the ΔΨ_*m*_ generated by l-Ser resulted in a ΔΨ_*m*_ of −190 mV, comparable to the positive control (l-Pro) and significantly different from the basal potential (~−90 mV) (Fig. 8B). These results show that converting l-Ser into pyruvate is the main (or maybe the only) metabolic pathway linking this amino acid to ATP production through OxPhos.

### Exometabolomics analysis to determine the excreted products from the metabolism of l-Ser

**Fig 9:**
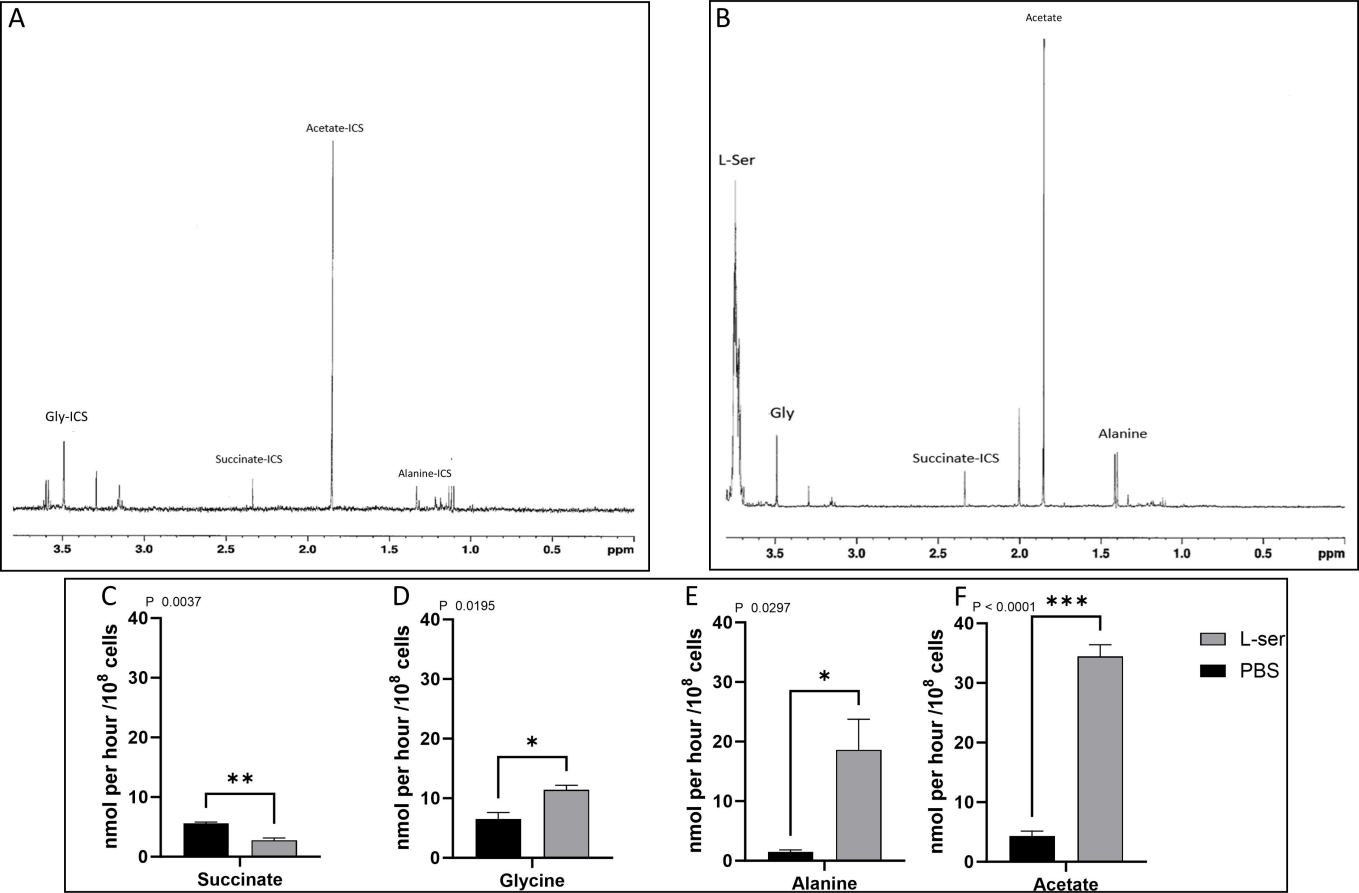
Excretion profile of metabolites from *T. cruzi* epimastigotes from l-Ser metabolism. (**A**) Proton resonance profile of metabolites excreted from parasites incubated for 6 h with PBS or (**B**) PBS supplemented with 5 mM l-Ser. (**C–F**) Quantification of identified metabolites. ICS, inner carbon sources. All data were shown as mean ± SD (*n* = 3). All experiments were replicated three times or more in three biological replicates. A two-tailed unpaired *t* test was used for statistical analysis. *P* < 0.05 was considered statistically significant.

Given the bioenergetic relevance of converting l-Ser into pyruvate, the metabolic pathways involved in these processes were investigated by analyzing the products excreted by these cells when exposed to this amino acid. The exometabolome was investigated by using quantitative ^1^H-NMR analysis. For this, cells were starved for 16 h and then recovered for 6 h in the presence (or not, PBS) of 5 mM l-Ser. The spectra of proton resonances from each sample’s last 6 h of incubation were analyzed and quantified (Fig. 9A and B).

**Fig 10:**
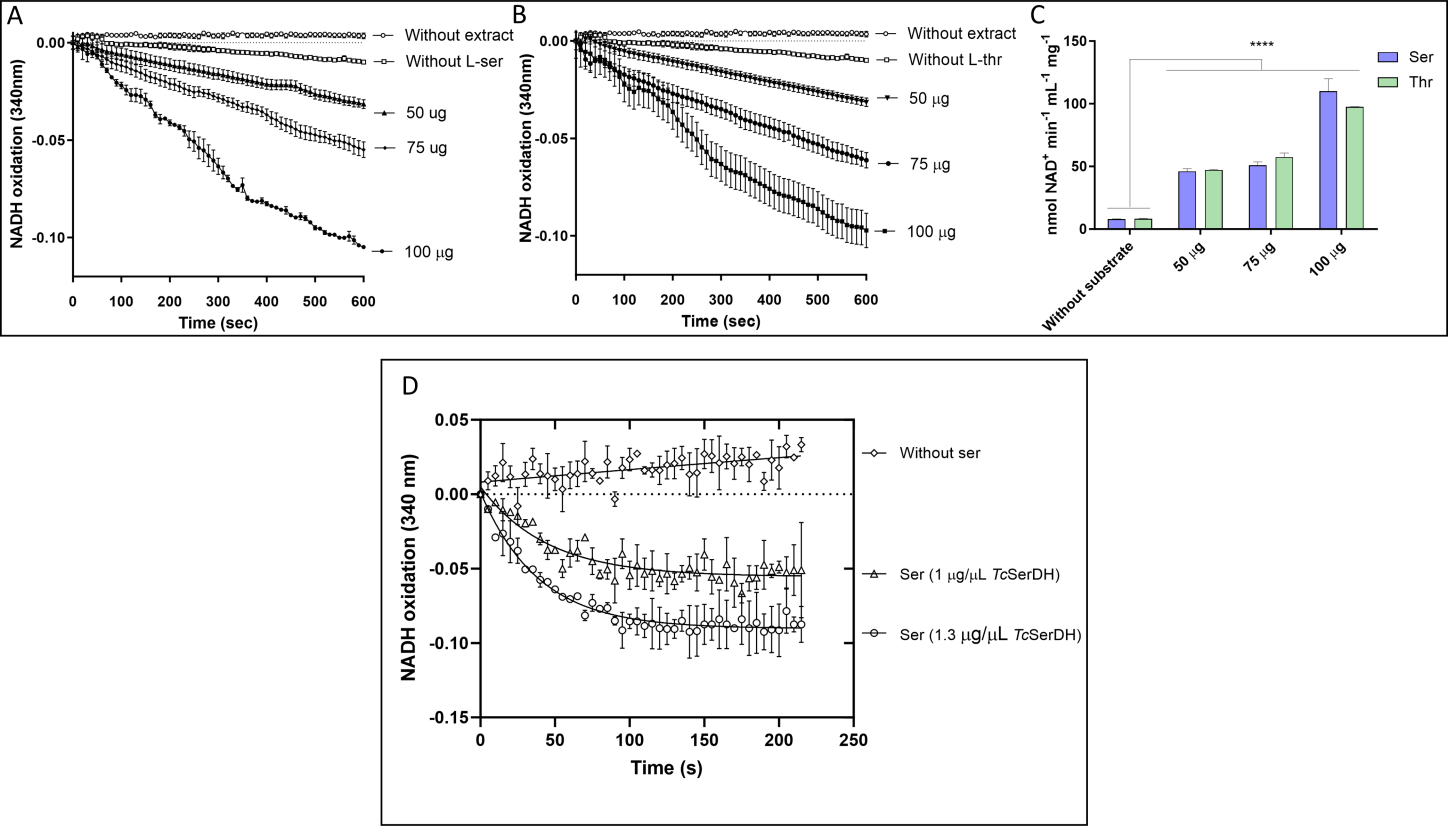
SerDH and ThrDH activities were measured by monitoring the decrease in absorbance at 340 nm (formation of NAD^+^) by coupling their reactions with recombinant lactate dehydrogenase (LDH). Briefly, l-SerDH produces pyruvate from l-Ser, which, through LDH, produces lactate and oxidizes NADH. For ThrDH activity, 2-oxobutyrate is produced, which through LDH produces 2-hydroxybutyrate, also oxidizing NADH. (**A**) Activity using l-Ser as a substrate in soluble extract of epimastigotes. (**B**) Activity using l-Thr as a substrate in soluble extract of epimastigotes. (**C**) NAD^+^ produced in the reaction at different concentrations of soluble parasite extract. (**D**) SerDH activity of the purified recombinant enzyme. All data were shown as mean ± SD (*n* = 3). All experiments were replicated three times or more in three biological replicates. Two-way ANOVA with Tukey post-test was used for statistical analysis. ****P < 0.0001.

Our results show that the main excretion products from l-Ser metabolism are, in order of quantity, Gly, Ala, acetate, and a decreased excretion of succinate compared with excretion products of parasites without exogenous carbon source (Fig. 9C through F, respectively). These data reinforce the idea that the metabolism of l-Ser in *T. cruzi* might occur through its conversion into pyruvate since both Ala and acetate are produced from pyruvate (45). A l-Ser/l-Thr dehydratase activity was detected in an epimastigote soluble extract (Fig. 10A through C). A search in the *T. cruzi* genome for an open reading frame encoding a putative Ser/Thr dehydratase was conducted to identify the molecular entity responsible for this activity. The identified gene (systematic number TcCLB.506825.70) was cloned in the pET-24a system, heterologously expressed in *Escherichia coli,* and the product fused to a C-terminal His6-tag was purified by Ni^2+^ affinity chromatography (Fig. S2). The purified recombinant protein showed an l-Ser/l-Thr dehydratase activity (Fig. 10D), confirming that the consumption of l-Ser for bioenergetics purposes in epimastigotes of *T. cruzi* can happen through its conversion into pyruvate.

## DISCUSSION

It has been shown that *T. cruzi*, after consuming preferentially glucose, can switch to the use of amino acids, mostly proline (43, 44, 46), but also glutamate, aspartate, glutamine (47), histidine (24), or fatty acids (9, 48, 49). The present work showed that l-Ser and l-Thr can also be used as primary carbon and energy sources. At the same time, Gly can participate in its metabolism by providing carbons but not energy. In most organisms, l-Ser can be produced *de novo* from 3-phosphoglycerate, a glycolytic/gluconeogenic intermediate (50, 51). However, *T. cruzi* lacks the last two steps of this pathway, suggesting that it relies on the uptake and/or an SHMT for obtaining l-Ser (22, 52). SHMT can form l-Ser by reverse reaction using Gly and 5,10-CH_2_-THF as substrates. Similarly, while plants, bacteria, and fungi can synthesize l-Thr *de novo* from aspartate (53, 54), *T. cruzi* lacks the first three steps of this pathway. It has been proposed that *T. cruzi*, like *T. brucei*, might be capable of synthesizing l-Thr from homoserine and acyl-homoserine lactones, constituting a “bypass” in the pathway at the homoserine kinase step (2.7.1.39) (19). Given these deficiencies in the biosynthesis pathways of l-Ser and l-Thr, *T. cruzi* relies on transport activities to obtain these molecules, as has been demonstrated in *T. brucei* (for l-Thr) (18) and *Leishmania amazonensis* (for l-Ser) (55).

### The uptake of l-Ser, l-Thr, and Gly occurs by a shared H^+^/ATP-driven transport system

The transport of metabolites can be considered the first step in metabolic pathways (10). Throughout its life cycle, *T. cruzi* is exposed to l-Ser, l-Thr, and Gly, which are present in the insect vector’s excreta (13, 14), as well as in mammalian cells and plasma (11, 12, 15). Many amino acid transport systems that have already been biochemically characterized in this organism (Table S1) have functional properties compatible with the AAAP family, which groups H^+^/amino acid transporters and auxin permeases (56, 57). Our data also show that the l-Thr, l-Ser, and Gly transport system characterized herein belongs to this group. Importantly, these data do not rule out the possibility of other specific or shared transport systems.

The *K*_*M*_ values for l-Ser and l-Thr are in the same range of values reported for Cys (58), Asp (59), and the low-affinity Arg transporters (60). The *K*_*M*_ value measured for Gly resembles those reported for l-Glu (61), l-Ile (23), l-His (24), and high-affinity l-Pro (system A) transporters (62). The *V*_max_ values obtained for l-Ser and l-Thr are within the range of those for the BCAA, l-His, l-Gln, and l-Pro low-affinity (system B) uptake systems (23, 24, 62, 63). Additionally, the *V*_max_ value for Gly is among the three highest ones reported for *T. cruzi*, together with l-Ala (64) and l-Leu (23). This could be related to the use of Gly as an osmolyte (65–68).

Significantly, the transport activity for l-Ser increased exponentially at temperatures between 25°C and 40°C, a range to which the parasites are naturally exposed within insect vectors and vertebrate hosts. This suggests that the environmental temperature might be a natural modulator of l-Ser, l-Thr, and Gly uptake. In addition, the *E*_*a*_ was the second lowest among those reported for amino acid transport systems in *T. cruzi*, after the low-affinity Arg transporter (60). Accordingly, from the *E*_*a*_ value, the equivalent of at least 1.6 molecules of ATP would be required per l-Ser molecule transported into the cells. Finally, our transport data show that this system is active, as shown for the transport of most of the other amino acids in *T. cruzi* (10). This contrasts with the l-Ser uptake in *L. amazonensis*, which appears to be directly powered by ATP (55). Noteworthy, our data showed that l-Ser uptake varied throughout the parasite’s life cycle, with the transient stage intracellular epimastigote showing the highest activity and the bloodstream trypomastigotes and metacyclics showing the lowest activities. This indicates different requirements for these amino acids during the parasite’s life cycle. l-Ser and l-Thr concentrations in plasma range from 100 to 200 µM in mammals, with values above the *K*_*M*_ measured for l-Ser and l-Thr transport (11, 12, 15). In triatomines, little is known about the abundance of these amino acids in different digestive tract compartments or excreta. However, it can be assumed that the epimastigote could find an environment rich in amino acids for two reasons: (i) during the blood meal, the triatomine has high proteolytic activity, efficiently breaking down whole blood proteins (mostly hemoglobin) and thus releasing free amino acids that the parasite could use; and (ii) during the bloodmeal, the triatomine usually releases amino acids into the intestinal lumen through the Malpighian tubules (13, 14).

### l-Ser and l-Thr are fully catabolized to CO_2_ and stimulate OxPhos in epimastigotes

Differently from Gly, l-Ser and l-Thr were catabolized to CO_2_ and could sustain the intracellular ATP levels and respiration when present as the only carbon source. It is worth noting that the study of the participation of Gly was motivated by the possible existence of two pathways leading to the production of CO_2_: (i) a SHMT activity that could interconvert Gly and 5,10-CH_2_-THF into l-Ser and THF (tetrahydrofolate) has been previously demonstrated in *T. cruzi* (21, 39); and (ii) the existence in the *T. cruzi* genome of putative genes encoding the glycine cleavage complex (without demonstration of enzymatic functionality so far). The absence of CO_2_ production from Gly rules out both possibilities in our experimental conditions. However, in *Leishmania major* promastigotes, it was suggested that Gly participates in one-carbon metabolism (39). Concerning l-Ser, the same work showed a critical decrease in *L. major* doubling time even in excess Gly (10-fold more) when l-Ser was removed from the semi-defined culture medium. This led to the conclusion that SHMT would not compensate for l-Ser demand (39). These results support the absence of classical *de novo* l-Ser biosynthesis pathways in trypanosomatids. In *T. brucei*, this complex may be essential since the parasite does not have SHMT and 10-formyl tetrahydrofolate synthetase annotated in its genome database, suggesting that it must rely exclusively on the glycine cleavage complex for folate metabolism. Finally, l-Ser carbon, which utilizes the MPC as its entry point, built up ΔΨ_*m*_ (42, 69). Accordingly, l-Ser may be converted into pyruvate in the cytosol and then transported into the mitochondrion by the MPC; or it could be transported into the mitochondrion as l-Ser by the MPC if this transporter has a broad specificity. In both cases, the l-Ser-derived pyruvate can supply intramitochondrial acetyl-CoA, which in turn can supply carbons into the TCA cycle, which has been shown to be functional in *T. cruzi* epimastigotes (8, 46, 70).

### The *Trypanosoma cruzi* epimastigotes have Ser and Thr dehydratase activities

In most eukaryotic cells, the catabolism of l-Ser happens through dehydration followed by deamination, producing pyruvate and NH_4_^+^ (71, 72) in the case of l-Ser and 2-oxobutanoate and NH_4_^+^ in the case of l-Thr (38). Both Ser/ThrDH activities were found in epimastigote soluble extracts. It is worth noting that l-Thr carbons can serve as a substrate for other putative enzymes predicted in the *T. cruzi* genome: a recently characterized l-Thr dehydrogenase (TDH) (73) and a 2-amino-3-ketobutyrate coenzyme A ligase (E.C. 2.3.1.29, TcCLB.511899.10), which have been demonstrated as crucial for acetate production and fatty-acid biosynthesis in procyclic form of *T. brucei* (20, 74). According to the results reported herein, the catabolism of l-Ser can contribute to the production of acetyl-CoA and acetate. Considering that (i) the mitochondrial acetate:succinate CoA transferase/succinyl-CoA synthetase (ASCT/SCS) cycle is operative in *T. cruzi* epimastigote (M. B. Alencar and A. M. Silber, unpublished data), and (ii) *T. cruzi* lacks putative genes for an acetyl-CoA hydrolase (ACH; EC: 3.1.2.1), the production of acetate from l-Ser and l-Thr could be coupled to substrate-level production of intramitochondrial ATP in addition to OxPhos. Furthermore, the excretion of Gly by l-Ser-fed parasites suggests that Gly is excreted as an osmotic control mechanism (66, 68).

**Fig 11:**
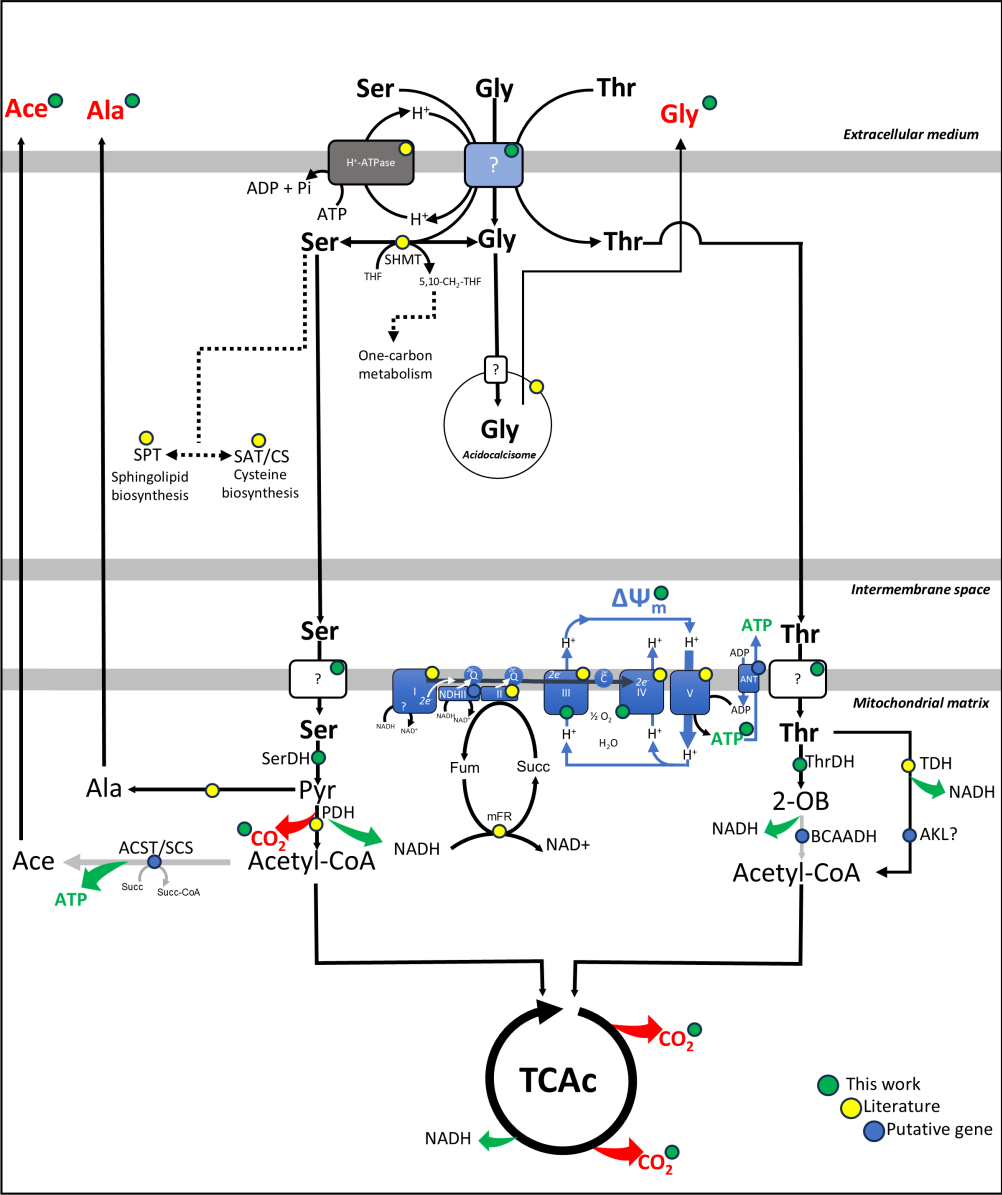
Metabolism of l-Ser and l-Thr in *Trypanosoma cruzi*. Metabolic steps are represented with different colors according to the origin of data: green-labeled steps correspond to data obtained in this work; yellow-labeled steps correspond to data obtained from the literature; blue-labeled steps correspond to inferred reactions according to annotations of the *T. cruzi* genome. In the light blue box, the same transport system for the three amino acids; in the gray box, plasma-membrane H^+^-ATPase (75); pyruvate dehydrogenase (PDH) (76); mitochondrial electron-transfer complexes (CI-CIV) (40, 77, 78); NADH-fumarate reductase (mFR) (79–81) (39, 76); F_1_F_o_-ATP synthase (40, 77); adenine nucleotide translocase (ANT) (TcCLB.511249.10) (82); SPT (TcCLB.506405.50) (9, 83); serine acetyltransferase and cysteine synthase (SAT/CS) (84, 85); SHMT (22); branched-chain alpha-keto acid dehydrogenase complex (BCAKDH) (Tc001047053506295.160; Tc001047053506853.50; Tc001047053507601.70; and Tc001047053507757.70);TDH (73); 2-amino-3-ketobutyrate coenzyme A ligase (AKL) (TcCLB.511899.10); ASCT (TcCLB.504153.360); SCS (α: TcCLB.508479.340 and β: TcCLB.507681.20).

In conclusion, l-Ser, l-Thr, and Gly can be taken up by the cells by a shared H^+^/ATP-dependent transport system and contribute to cellular homeostasis maintenance during nutritional stress, providing conditions necessary for the resumption of epimastigote proliferation. l-Ser and l-Thr can be further oxidized with the production of CO_2_, triggering O_2_ consumption, contributing to the maintenance of the inner mitochondrial membrane potential, and powering ATP production through OxPhos. With the evidence collected throughout this work and following the data collected in the literature and genetic databases, we propose a model for the energy metabolism of l-Ser and l-Thr, as shown in Fig. 11.

## MATERIALS AND METHODS

### Reagents

l-[3-^14^C]Ser, l-[U-^14^C]Thr, and [U-^14^C]Gly (0.1 mCi/mL) were purchased from American Radiolabeled Chemicals, Inc. (St. Louis, MO, USA). All other reagents were from Sigma (St. Louis, MO, USA).

### Parasites

*T. cruzi* CL strain clone 14 epimastigotes were maintained in the exponential growth phase by subculturing them every 48 h in liver infusion tryptose (LIT) medium supplemented with 10% fetal calf serum at 28°C (86). The other developmental forms were acquired by adhering to the methodologies outlined by Silber et al. (87,87) and Damasceno et al. (63). Briefly, to obtain metacyclic trypomastigote forms, stationary phase epimastigotes (5 × 10^7^ cells/mL) were submitted to nutritional stress in TAU medium for 2 h and then transferred to TAU3 AAG to initiate differentiation (88). After 6 days, metacyclic trypomastigotes were purified using a DEAE-cellulose resin as previously described obtained by *in vitro* differentiation in a defined medium (TAU3 AAG) as described previously (89). Bloodstream trypomastigotes were obtained from CHO-K_1_ infection as described in reference 90. Amastigotes and intracellular epimastigotes (29) were obtained by lysing the host cells 48 and 80 h post-infection, respectively. The infected host cells were washed with PBS and lysed using 0.05% SDS. The lysates were monitored by microscopy and halted with the addition of 10% SFB in PBS. The parasites were separated from cellular debris through two centrifugation steps: the first involved centrifugation for 10 min at 115 × *g* and 4°C, the supernatant was recovered, followed by centrifugation for 10 min at 2,900 × *g* and 4°C. The parasites were then collected from the pellets. The purity of the intracellular forms was assessed microscopically, and the yield was determined in a Neubauer chamber.

### Transport assays

Transport assays were performed as described previously (23, 24, 62–64). Briefly, transport assays were initiated by the addition of 100 µL of 5 mM l-Ser, l-Thr, or Gly in PBS to aliquots of parasites of 100 µL (2 × 10^7^ cells each, except when otherwise specified), traced with 0.4 µCi of radiolabeled amino acid. The uptake was measured at 28°C for 3 min, except when otherwise specified. The transport reaction was stopped by the addition of 800 µL of stop solution (50 mM amino acid in PBS, pH 7.2), prechilled at 4°C, immediately followed by two washes with cold PBS (10,000 × *g,* 2 min, and 4°C). Background values in each experiment were measured by the simultaneous addition of each traced amino acid and stop solution. The parasites were resuspended in 50 µL of PBS and transferred to scintillation fluid. The samples were measured in a Perkin Elmer Tri-Carb 2910 TR scintillation detector. For competition assays, l-Ser, l-Thr, or Gly uptake was measured at concentrations equivalent to the *K*_*M*_ (37, 87, and 260 µM for l-Ser, l-Thr, and Gly, respectively) in the presence of 10 times the *K*_*M*_ of each other amino acid. For assessing the influence of the plasma membrane proton gradient on the l-Ser uptake, parasites were treated with 10 µM carbonyl cyanide *m*-chlorophenyl hydrazone. As previously reported, protonophore treatment can affect an uptake process due to the disruption of the H^+^ gradient across cellular membranes (if the uptake is performed through an H^+^/metabolite symporter) or the decrease of intracellular levels of ATP due to its rapid consumption by the mitochondrial F_1_F_o_-ATP synthase, which in a low-mitochondrial-membrane-potential situation hydrolyzes ATP to pump H^+^ to reestablish the ∆Ψ_*m*_ (25–28, 91). To discriminate between both effects, a control was performed by adding 5 µg/mL oligomycin A to CCCP-treated cells, which allowed simultaneous disruption of H^+^ membrane gradients while blocking the F_1_F_o_-ATPase. The ATP-dependence assay was conducted as reported in references 23, 24, 63.

After obtaining the different forms as described in references 63, 87, the cells were resuspended in PBS and counted using a Neubauer chamber. The cell density was adjusted to a final density of 20 × 10^7^ cells/mL and distributed in aliquots of 100 µL (2 × 10^7^ cells each). The initial velocity (*V*_0_) of the incorporation was measured at 28°C (Epi and MTrypo) and 37°C (Ama, Epi-like, and BTrypo) for 3 min.

### Analysis of transport assay data

The disintegrations per minute (dpm) corresponding to transported radiolabeled amino acid for each experimental point (dpmi) were calculated as equation 1:


(1) dpmi=dpme−dpmb,

where dpme is the average dpm from triplicates after 1 min of incubation in the presence of radiolabeled amino acid, and dpmb is the average dpm from the background samples.

The amount of amino acid taken up by the cells was calculated as equation 2:


(2) $$AAi=dpmi\;\left[AA\right]\;v\;{dpmst}^{-1}\;t^{-1},$$

where AAi is the transported amino acid, [AA] is the amino acid nanomolar concentration, *v* is the volume of radiolabeled amino acid solution in the experiment, dpmst is the total dpm measured for each added radiolabeled amino acid, and *t* is the time of incubation measured in minutes.

### Viability and proliferation resumption assays

Epimastigotes (5 × 10^7^ cells) were incubated for 24, 48, 72, and 96 h at 28°C in PBS supplemented or not with 5 mM l-Ser, l-Thr, Gly, or histidine (as a positive control for the maintenance of the viability) to induce cell recovery. After recovery, the cells were washed in PBS and incubated with resazurin reagent to evaluate cell viability, as previously described (92) or transferred to LIT medium at a final density of 2.5 × 10^6^ parasites/mL and then incubated (200 µL per well in experimental triplicates for each biological replicate) in 96-well plates at 28°C to assess their capacity of resuming proliferation. The plates were read using an ELx800 Absorbance Reader (Bio Tek), where the dispersion of light by the culture was measured at *λ*_620_. Using a calibration curve with known parasite concentrations, assay values of light scattering by the culture (optical density, OD) were converted to number of parasites/mL.

### Quantification of ΔΨ_*m*_

Epimastigotes (~4–5 × 10^7^ parasites/mL) were centrifuged at 1,000 × *g* for 10 min at 4°C and washed twice in ΔΨ_*m*_ buffer (250 mM sucrose, 10 mM HEPES, 250 µM EGTA, 2 mM NaH_2_PO_4_, and 1 mM MgCl_2_ pH 7.2). Cells were adjusted to a density of 1 × 10^9^ parasites/mL. Aliquots of 50 µL were separated and permeabilized in the presence of 5 µM digitonin for 30 min. Safranin fluorescence was measured (EX WL: 495.0 nm and EM WL: 586.0 nm) in a cuvette fluorometer (F-7100 FL Spectrophotometer Hitachi High-Tech). Cells (5 × 10^7^ cells) were added to the cuvette in 2 mL ΔΨ_*m*_ buffer, 0.1% BSA (free of fatty acids), 12.5 µM safranin, and 5 µM digitonin. The calibration curve was constructed under the same conditions as mentioned before. Then, 5 nM valinomycin was added, followed by titration using KCl. Finally, 1.25 µM FCCP was added to uncouple the ΔΨ_*m*_ fully. The values were adjusted to the Nernst equation to estimate ΔΨ_*m*_.


$$Nernst\;equation:\Delta \Psi_{m}=60\times \log\left(K_{IN}{}^{+}/K_{OUT}{}^{+}\right).$$

### Quantification of excreted metabolites by ^1^H-NMR analysis

Epimastigotes (1 × 10^8^/mL) were collected by centrifugation at 1,400 × *g* for 10 min, washed twice with PBS, and incubated in 1 mL of PBS supplemented with 25 mg/mL NaHCO_3_ (pH 7.2). The cells were starved for 16 h in PBS and for 6 h with 5 mM l-Ser at 28°C or no exogenous carbon sources. The integrity of the cells during the incubation was checked by microscopic observation. The supernatant (1 mL) was collected and lyophilized (SpeedVac SPD1030). The samples were reconstituted, and 50 µL of maleate solution in D_2_O (10 mM) was added as an internal reference. ^1^H-NMR spectra were collected at 500.19 MHz on a Bruker Avance III 500 HD spectrometer with a 5 mm Prodigy cryoprobe. The measurements were recorded at 25°C. The acquisition conditions were as follows: 90° flip angle, 5,000 Hz spectral width, 32 K memory size, and 9.3 s total recycling time. The measurements were performed with 64 scans for a total time of close to 10 min 30 s. The resonances of the obtained spectra were integrated, and the metabolite concentrations were calculated using the ERETIC2 NMR quantification Bruker program.

### ATP recovery using l-Ser, l-Thr, and Gly

The parasites (approximately 5 × 10^7^ cells/mL) were starved as described above and recovered or not (negative control) by incubation for 1 h in the presence of 5 mM His (as positive controls) or 5 mM l-Ser, l-Thr, or Gly. The intracellular ATP concentration in each sample was determined after recovery using a luciferase assay according to the manufacturer’s instructions (Sigma). ATP concentrations were estimated by using a calibration curve; luminescence (*λ*_570_ nm) was detected using a SpectraMax i3 plate reader (Molecular Devices, Sunnyvale, CA, USA).

### CO_2_ production measurements

Epimastigotes (5 × 10^7^ parasites/mL) were washed twice, resuspended in PBS, and incubated in 5 mM l-Ser, l-Thr, or Gly spiked with 0.1 µCi of radiolabeled amino acid for 0.5, 1, 2, 3, and 4 h at 28°C. To trap the produced CO_2_, pieces of Whatman filter soaked in 2 M KOH were placed on the top of the tubes where the parasites were incubated. The filters were incubated with a scintillation cocktail, and the K_2_^14^CO_3_ trapped on the paper was measured by using a scintillation counter. To assess the comparative release of carbon as CO_2_, the measured values were normalized against the quantity of the transported amino acid over an identical incubation period.

### Oxygen consumption

Epimastigotes (5 × 10^7^ cells/mL) were nutritionally stressed for 16 h in PBS at 28°C and recovered or not (negative control) for 3 h at 28°C in the presence of 5 mM His (positive control) or 5 mM l-Ser, l-Thr, and Gly as the only exogenous carbon sources. The parasites were added to the respiration buffer (MCR: 125 mM sucrose, 65 mM KCl, 10 mM HEPES NaOH, 1 mM MgCl_2_, and 2 mM K_2_HPO_4_, pH 7.2). Subsequently, oligomycin A and FCCP were sequentially titrated. A mitochondrial complex III inhibitor, antimycin A, was added to verify residual respiration. Oxygen consumption rates were measured using intact cells in the high-resolution Oxygraph (OROBOROS, Oxygraph-2k, Innsbruck, Austria). Data were recorded and treated with DataLab 8 software.

### Expression and purification of recombinant *Tc*Ser/ThrDH

For the expression of Ser/ThrDHr, *E. coli* BL21 Codon-Plus containing the pET24a-TcSer/ThrDH plasmid, producing the product fused to a C-terminal His6-tag, were incubated (37°C and 180 rpm) in 1 L of LB medium with kanamycin (30 µg/mL) and tetracycline (5 µg/mL). Once they reached an optical density (OD 600 nm) of 0.5, 0.1 mM IPTG and 100 µM PLP were added to the culture, followed by incubation at 37°C for 16 h (180 rpm). The cells were then harvested and centrifuged (15 min, 5,000 × *g*, 4°C), and the pellet was resuspended in binding buffer (20 mM Tris-HCl [pH 7.4], 500 mM NaCl, and 10 mM imidazole). The bacteria were lysed by treatment with lysozyme (Sigma) at 1 mg/mL (30 min at 4°C) and six pulses of sonication (six pulses of 20 s with 20% amplitude and 20 s intervals). The lysate was clarified by centrifugation (30 min, 16,000 × *g* at 4°C). For the purification of the recombinant enzyme, the soluble fraction derived from the culture expressing *Tc*Ser/ThrDHr was subjected to nickel affinity chromatography (Ni^2+^-NTA agarose-Qiagen) following the manufacturer’s instructions. The recombinant protein was eluted in buffer with 500 mM imidazole, quantified by the Bradford method, and dialyzed in 5 L of PBS with 5% glycerol and 100 µM PLP for 16 h at 4°C. The purification was analyzed using 10% SDS-PAGE.

### Statistical analysis

Curve adjustments, regressions, standard deviation, and statistical analysis were performed with the GraphPad Prism 10 analysis tools. All assays were performed at least in biological triplicate, and the details of statistical analysis were added to each figure legend.

## SUPPLEMENTAL MATERIAL

Text S1 msphere.00983-24-s0001.docx Calculations.

Figure S1 msphere.00983-24-s0002.docx Assessment of membrane potential loss using CCCP and ATP decrease by oligomycin A treatment.

Figure S2 msphere.00983-24-s0003.docx Optimization of the expression of the recombinant serine dehydratase of *Trypanosoma cruzi*.

Table S1 msphere.00983-24-s0004.docx Overview of the biochemical characterization of amino acid transport systems to date in *Trypanosoma cruzi*.

ASM does not own the copyrights to Supplemental Material that may be linked to, or accessed through, an article. The authors have granted ASM a non-exclusive, world-wide license to publish the Supplemental Material files. Please contact the corresponding author directly for reuse.
