# Supplementary material for: The role of l-serine and l-threonine in the energy metabolism and nutritional stress response of Trypanosoma cruzi
Source: mSphere. 2025 Mar 5;10(3):e00983-24. doi: 10.1128/msphere.00983-24 (PMC11934319; doi:10.1128/msphere.00983-24)
Supplement: Text S1 — Calculations. [file msphere.00983-24-s0001.docx]

**Supplementary material**

**Text S1: Calculations**

Computation:

*K*_cat_ = ${Ae}^{-\frac{E_{a}}{RT}}$

At 30°C, [S] at saturate concentration

R has the value of 8.3144621 x 10^-3^ kJ mol^-1^K^-1^

A = 1.34 x 10^16^ (from the Arrhenius Equation plot)

E = natural log base (2.718281828459)

*E*_a_ = 51.23 kJ/mol

51,23 => kcat **1.995 x 10^7^ molecules min^-1^  = 1.197 x 10^9^ molecules s^-1^**

T = 303.15 K

*K*_cat_ = ${Ae}^{-\frac{E_{a}}{RT}}$

*K*_cat_ Value obtained **1.995 x 10^7^ molecules min^-1^  = 1.197 x 10^9^ molecules s^-1^**

*V*_max_ = 0.258 nmol/min per 20x10^6^ cells

no. of transporters: $Et=\frac{V_{max}}{{Ae}^{-\frac{E_{a}}{RT}}}$ = $\frac{V_{max}}{K_{cat}}$

Et = $\frac{V_{max}}{K_{cat}}$ = 0.258/0.00198 = 0.00198 nmol per 20.10^6^ cells

= 1.99 x10^-12^ mol x 20.10^6^ cells

= 9.9 x10^-13^ mol x 10.10^6^ cells

= 9.94x 10^-5^ amol x 10.10^6^ cells

Average: 0.09938325 amol per cell

5.98 x 10^4^ active sites per cell
