# Supplementary material for: The role of l-serine and l-threonine in the energy metabolism and nutritional stress response of Trypanosoma cruzi
Source: mSphere. 2025 Mar 5;10(3):e00983-24. doi: 10.1128/msphere.00983-24 (PMC11934319; doi:10.1128/msphere.00983-24)
Supplement: Figure S1 — Assessment of membrane potential loss using CCCP and ATP decrease by oligomycin A treatment. [file msphere.00983-24-s0002.docx]

**Figure S1. Assessment of membrane potential loss using CCCP and ATP decrease by Oligomycin A treatment**

A luciferase assay was performed to verify the decrease in the intracellular ATP levels under treatment with Oligomycin A. Briefly, the intracellular ATP concentration in each sample was determined after 30 min using a luciferase assay according to the manufacturer’s instructions (Sigma). ATP concentrations were estimated by using a calibration curve; luminescence (λ570 nm) was detected using a SpectraMax i3 plate reader (Molecular Devices, Sunnyvale, CA). The treatment with 5 µg/mL Oligomycin A induces a 72.3% decrease in the intracellular ATP levels (S1 A).

To assess the ability of CCCP to induce the loss of proton gradient, parasites (5 x 10^7^ cells per ml) were washed twice in PBS and incubated (or not) with 10 µM CCCP. The unlabeled cells were used as a positive control for loss of membrane potential. Then, the parasites were incubated with 250 nM Rh123 for 20 min at 28 °C, washed with cytomix buffer (25 mM HEPES-KOH buffer, 120 mM KCl, 0.15 mM CaCl_2_, 2 mM EDTA, 5 mM MgCl_2_, 10 mM K^+^-phosphate buffer and 10 μM CCCP) and 10.000 events were analyzed in the FL-1 detector of a FACS-Calibur flow cytometer.

The treatment with 10 µM CCCP induces a complete loss of membrane potential in the cell population, while cells in PBS (not starved) present high fluorescence levels (S1. B).


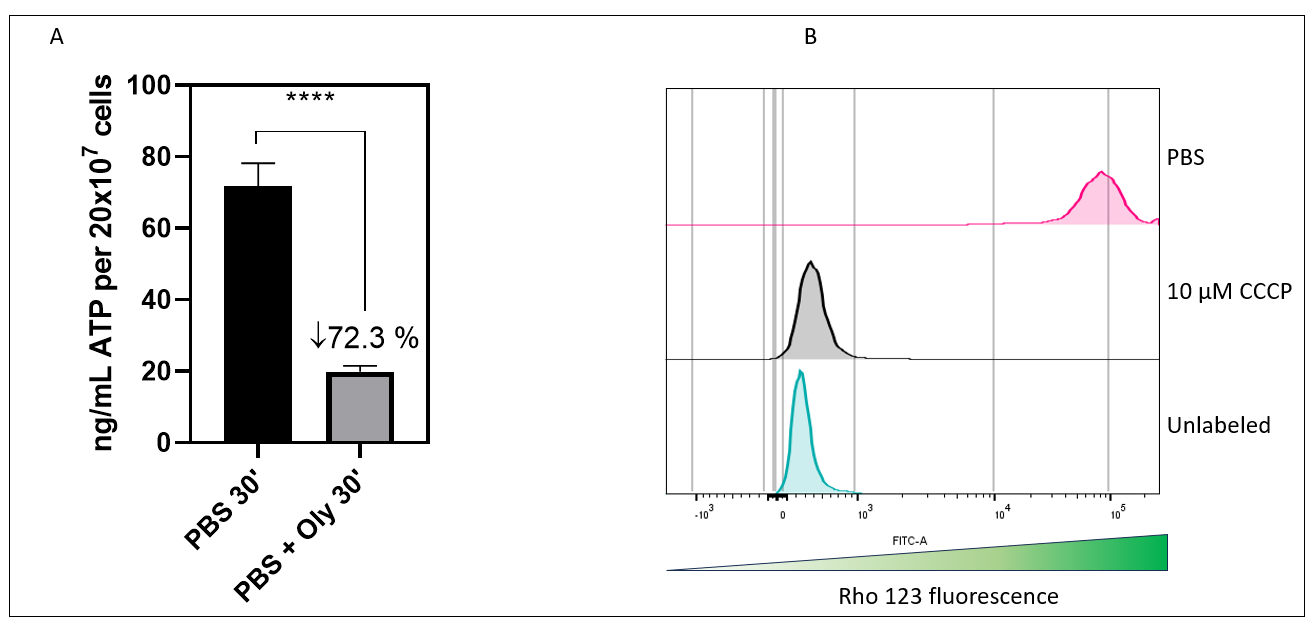


S1. (A) Quantification of cytoplasmic ATP levels assessed using a bioluminescent assay; (B) Representative histograms were obtained for the labeled and unlabelled cells in each experimental condition. Cells were treated or not (control) with 10 μM CCCP, and the Rhodamine 123 fluorescence in the cells was analyzed by flow cytometry. In total, 10,000 events were analyzed for each sample. The data were shown as mean ± SD (n =3). All experiments were replicated three times or more in three biological replicates and t test was used for statistical analysis. **** P< 0.0001.
