# Supplementary material for: The role of l-serine and l-threonine in the energy metabolism and nutritional stress response of Trypanosoma cruzi
Source: mSphere. 2025 Mar 5;10(3):e00983-24. doi: 10.1128/msphere.00983-24 (PMC11934319; doi:10.1128/msphere.00983-24)
Supplement: Figure S2 — Optimization of the expression of the recombinant serine dehydratase of Trypanosoma cruzi. [file msphere.00983-24-s0003.docx]

**Figure S2. Optimization of the expression of the recombinant Serine Dehydratase of *Trypanosoma cruzi*.**


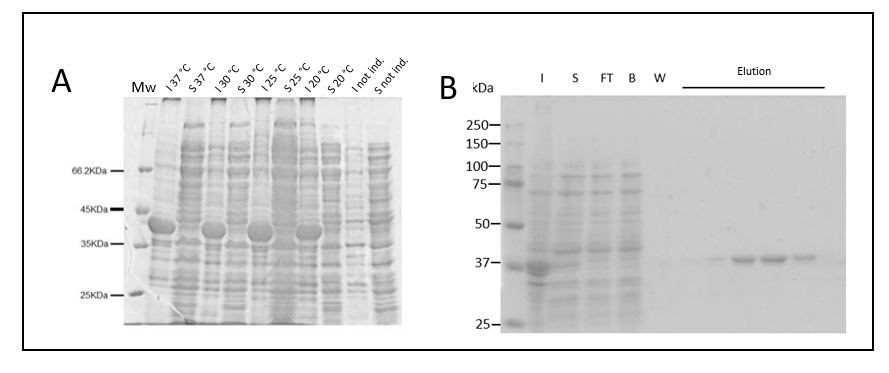


S2. (A) induction of the recombinant protein as a function of temperature. (B) SDS-PAGE of the recombinant enzyme purification from E. coli BL21 Codon Plus extracts by affinity chromatography on a Ni2+-NTA column; I: insoluble; S: Soluble; FT: Flow Trough; B: Binding; W: Wash.
