# Supplementary material for: The role of l-serine and l-threonine in the energy metabolism and nutritional stress response of Trypanosoma cruzi
Source: mSphere. 2025 Mar 5;10(3):e00983-24. doi: 10.1128/msphere.00983-24 (PMC11934319; doi:10.1128/msphere.00983-24)
Supplement: Table S1 — Overview of the biochemical characterization of amino acid transport systems to date in Trypanosoma cruzi. [file msphere.00983-24-s0004.docx]

**Table S1. Overview of the biochemical characterization of amino acid transport systems to date in *Trypanosoma cruzi*.**

|  |  |  |  | ***Ion and ATP dependence*** | | | |  |
| --- | --- | --- | --- | --- | --- | --- | --- | --- |
| ***Amino acid*** | ***K_M_ (mM)*** | ***V_max_ (nmol·min^−1^ per 20 × 10^6^ cells)*** | ***E_a_ (kJ/mol)*** | ***H^+^*** | ***K^+^*** | ***Na^+^*** | ***ATP*** | ***Ref.*** |
| l-Alanine | 1.81 | 1.86 | 66.4 | yes | no | no | yes | (3) |
| l-Histidine | 0.12 | 0.24 | 55.25 | no | no | no | yes | (32) |
| l-Leucine | 1.07 | 0.94 | 51.3 | yes | no | no | yes | (5) |
| l-Isoleucine | 0.47 | 0.16 |  |  |  |  |  |  |
| l-Valine | 1.96 | 0.33 |  |  |  |  |  |  |
| l-Glutamine | 1.29 | 0.36 | 63.29 | yes | no | no | yes | (2) |
| l-Glutamate | 0.3 | 0.09834 | 52.38 | yes | no | no | yes | (6) |
| l-Arginine | 0.0485 | 0.03466 | 31.1 | ND | ND | ND | yes | (7,8) |
| lAspartate | 0.032 | 0.0068 | ND | ND | ND | ND | ND | (9) |
| l-Cysteine | 0.0495 | 0.026 | ND | ND | ND | ND | ND | (10) |
| l-Proline A | 0.31 | 0.012 | 79.5 | yes | no | no | yes | (11) |
| l-Proline B | 1.36 | 0.31 | 18.3 e 176.5 | yes | no | no | yes |  |
| Glycine | 0.267 | 0.902 | 51.23 | yes | ND | ND | yes |  |
| l-Serine | 0.037 | 0.258 |  | yes | ND | ND | yes | This work |
| l-Threonine | 0.087 | 0.367 |  | yes | ND | ND | yes |  |

ND: no data

**References**

1. Silber AM, Tonelli RR, Lopes CG, Cunha-e-Silva N, Torrecilhas ACT, Schumacher RI, et al. Glucose uptake in the mammalian stages of Trypanosoma cruzi. Mol Biochem Parasitol. 2009 Nov;168(1).

2. Damasceno FS, Barisón MJ, Crispim M, Souza ROO, Marchese L, Silber AM. L-Glutamine uptake is developmentally regulated and is involved in metacyclogenesis in Trypanosoma cruzi. Mol Biochem Parasitol. 2018;224.

3. Girard RMBM, Crispim M, Alencar MB, Silber AM. Uptake of l-Alanine and Its Distinct Roles in the Bioenergetics of Trypanosoma cruzi. mSphere. 2018;3(4).

4. Barisón MJ, Damasceno FS, Mantilla BS, Silber AM. The active transport of histidine and its role in ATP production in Trypanosoma cruzi. J Bioenerg Biomembr. 2016;48(4):437–49.

5. Manchola NC, Rapado LN, Barisón MJ, Silber AM. Biochemical Characterization of Branched Chain Amino Acids Uptake in Trypanosoma cruzi. J Eukaryot Microbiol. 2016;63(3).

6. Silber AM, Rojas RLG, Urias U, Colli W, Alves MJM. Biochemical characterization of the glutamate transport in Trypanosoma cruzi. Int J Parasitol. 2006 Feb;36(2):157–63.

7. Hampton JR. Arginine transport in the culture form of Trypanosoma cruzi. J Protozool. 1971 Nov;18(4):701–3.

8. Pereira CA, Alonso GD, Paveto MC, Flawiá MM, Torres HN. L-arginine uptake and L-phosphoarginine synthesis in Trypanosoma cruzi. J Eukaryot Microbiol. 1999;46(6).

9. Canepa GE, Bouvier LA, Urias U, Miranda MR, Colli W, Alves MJM, et al. Aspartate transport and metabolism in the protozoan parasite Trypanosoma cruzi. FEMS Microbiol Lett. 2005 Jun 1;247(1):65–71.

10. Canepa GE, Bouvier LA, Miranda MR, Uttaro AD, Pereira CA. Characterization of Trypanosoma cruzi L-cysteine transport mechanisms and their adaptive regulation. FEMS Microbiol Lett. 2009 Mar;292(1).

11. Silber AM, Tonelli RR, Martinelli M, Colli W, Alves MJM. Active transport of L-proline in Trypanosoma cruzi. J Eukaryot Microbiol. 2002;49(6):441–6.
